# Hot Deformation Characteristics—Constitutive Equation and Processing Maps—of 21-4N Heat-Resistant Steel

**DOI:** 10.3390/ma12010089

**Published:** 2018-12-27

**Authors:** Yiming Li, Hongchao Ji, Wangda Li, Yaogang Li, Weichi Pei, Jinping Liu

**Affiliations:** 1 College of Mechanical Engineering, North China University of Science and Technology, Tangshan 063210, China; jxlym666@163.com (Y.L.); lwd13731542189@163.com (W.L.); jxlyg@ncst.edu.cn (Y.L.); pwc@ncst.edu.cn (W.P.); 2 National Center for Materials Service Safety, University of Science and Technology Beijing, Beijing 100083, China; 3 School of Mechanical Engineering, University of Science and Technology Beijing, Beijing 100083, China

**Keywords:** 21-4N heat-resistant steel, hot deformation, constitutive equation, processing map, microstructure

## Abstract

The hot deformation behavior of 21-4N heat-resistant steel was studied by hot compression test in a deformation temperature range of 1000–1180 °C, a strain rate range of 0.01–10 s^−1^ and a deformation degree of 60%, and the stress-strain curves were obtained. The functional relationship between flow stress and process parameters (deformation degree, deformation temperature, strain rate, etc.) of 21-4N heat-resistant steel during hot deformation was explored, the constitutive equation of peak stress was established, and its accuracy was verified. Based on the dynamic material model, the energy dissipation maps and destabilization maps of 21-4N heat-resistant steel were established at strains of 0.2, 0.4 and 0.6, and processing maps were obtained by their superposition. Within the deformation temperature range of 1060~1120°C and a strain rate range of 0.01–0.1 s^−1^, there is a stable domain with the peak efficiency of about 0.5. The best hot working parameters (strain rate and deformation temperature) of 21-4N heat-resistant steel are determined by the stable and instable domain in the processing maps, which are in the deformation temperature range of 1120–1180 °C and the strain rate range of 0.01–10 s^−1^.

## 1. Introduction

21-4N is an austenitic heat-resistant steel used in very hostile environments, such as those with high temperature, high pressure and corrosive gas. It is characterized by high tensile strength and yield strength, good oxidation resistance, excellent chemical stability and good high-temperature mechanical properties, and is widely used in the manufacture of engine valves [1,2,3,4,5]. The mechanical properties of 21-4N heat-resistant steel at high temperature are derived from the heat treatment process; solution treatment and aging treatment have a significant effect on enhancing its strength and corrosion resistance [6,7,8]. The steel can be resistant; Ni in steel can guarantee and improve its corrosion resistance, and a high content of Cr can ensure that forgings are free from oxidation in extreme environments [9,10]. At present, studies on 21-4N heat-resistant steel are mainly focused on improving its corrosion resistance, abrasive resistance, chemical stability, organizational properties and carbide precipitation [11,12,13]. However, a few studies have been carried out investigating the manufacturing technologies for 21-4N heat-resistant steel, especially with regard to the constitutive relation and processing map, as there are still many difficulties in determining the optimum hot working parameters. The research into the constitutive relation is helpful to describe the relationship between the dynamic response and hot working parameters and provide reference for the metal plastic process. The research into processing maps is helpful to describe the influence of hot working parameters on metal flow in the forming process, in order to avoid the generation of defects and achieve the purpose of controlling microstructure and performance.

In the past few years, many scholars have carried out lots of studies on constitutive relations and processing maps of different metal alloys. Ji et al. [14] conducted hot deformation compression testing on M_50_NiL alloy using a Gleeble-3500 thermal simulation test machine, established Arrhenius constitutive equation by using stress-strain data, and used a neural network to compensate the material constant. Hao et al. [15] studied the plastic deformation behavior of nickel-based superalloy 718 at strain rate of 5 × 10^3^–10.5 × 10^3^ s^−1^ and deformation temperature of 500–800 °C, and established the complex rheological stress constitutive equation to describe the processing hardening and softening in plastic deformation behavior. Li et al. [16] studied the mechanical properties of 25CrMo4 steel, established the constitutive equation by using the stress-strain data obtained from quasi-static and stamping tests, and verified the accuracy based on the structural variation of the grain centroid cubic and face-centered cubic of 25CrMo4 steel, which provided a reference for practical engineering analysis. Zhao et al. [17] established visco-plastic constitutive equation based on the principle of mechanics to predict the visco-plastic flow of TA15 alloy in plastic deformation. Cheng et al. [18] studied the hot deformation behavior of Ti-25Al-14Nb-2Mo-1Fe alloy in deformation temperature range of 950–1100 °C and strain rate range of 0.001–1 s^−1^ with the maximum strain of 50% by hot deformation compression test, analyzed the variation characteristics of the stress-strain curve, and determined material parameters such as stress index and deformation activation energy. Wu et al. [19,20] established constitutive equation by using stress-strain date that obtained from hot deformation compression test of AZ61 magnesium alloy and analyzed the relationship between constitutive parameters and rheological behavior. Mei et al. [21] studied the hot deformation behavior of AZ91 magnesium alloy in the deformation temperature range of 437–623 K and the strain rate range of 0.001–1 s^−1^ through unidirectional compression test and proposed a new method to describe constitutive equation and calculate the material constants K, C_1_ and C_2_ by using a piecewise function model. Liu et al. [22] studied the hot deformation behavior of LZ91 magnesium alloy. The constitutive equation was established by calculating its activation energy and material constant, and its phase transition characteristics during deformation were analyzed. Sui et al. [23] studied the hot deformation behavior of Inconel 718 alloy in the strain rate range of 0.001–100 s^−1^ and the deformation temperature range of 950–1150 °C, and obtained processing maps at strains of 0.1, 0.3, 0.5 and 0.7. Kong et al. [24] studied the hot deformation behavior of Ni-based C276 high-temperature alloy, established processing maps based on stress and strain data that obtained in test, analyzed stable domain and instability domain, and determined optimum hot working parameters. Wang et al. [25] studied the influence of the concentration of Al_2_O_3_ on the microstructure and mechanical properties of tungsten-based alloys, determined the optimum concentration of 0.25 wt.%, and calculated its thermal deformation activation energy and constitutive equation. Lu et al. [26] studied the rheological stress of 7075 aluminum alloy at different temperatures and strain rates, established processing maps, and obtained the optimum hot working parameters—(1) the deformation temperature range of 573–680 K and strain rate range of 0.368–8.1 s^–1^, and (2) the deformation temperature range of 695–723 K and strain rate range of 0.05–1 s^–1^—and the results provided a reference for sheet forming process design. Meng et al. [27] studied the stress-strain relationship of ATI425 titanium alloy using a Gleeble-3500 thermal simulation test machine. By modifying parameters such as strain, deformation temperature and strain rate, they established the constitutive equation and processing maps. Chen [28] studied the dynamic recrystallization behavior of Al-Zn-Mg alloy, established constitutive equation based on the improved Arrhenius and Johnson-Cook models, and verified the accuracy of the simulation results by extrusion experiments. Cheng et al. [29] studied the hot deformation behavior and softening mechanism of Mg-8Sn-2Zn-2Al alloy, analyzed the influence of temperature and strain rate on work hardening, and established the heat processing maps with strain of 0.1–0.7 to determine the best thermal processing parameters. Gong et al. [30] carried out isothermal deformation compression tests on 34CrNiMo6 alloy steel in the strain rate range of 0.005–1 s^−1^ and the deformation temperature range of 1173–1473 K using a Gleeble-1500 thermal simulation test machine, and obtained the stress-strain curves, established the constitutive equations and processing maps, and determined the optimum hot working parameters. Shi et al. [31] studied the hot deformation behavior of GH494 super-alloy, established flow stress constitutive equation with the deformation activation energy of 458.446 kJ·mol^−1^, made a processing map at strains of 0.2, 0.4, 0.6, and determined the optimum hot working parameters: 1082–1131 °C, 0.004–0.018 s^−1^; and 1134–1150 °C, 0.018–0.213 s^−1^. Kishor et al. [32] used the results of the hot deformation compression test of 16Cr-5Ni stainless steel to establish constitutive equation and processing maps, and determined the optimum hot working parameters: the deformation temperature range of 1000–1100 °C and strain rate range of 0.01–0.1 s^–1^, with a peak efficiency η of about 31%. Most studies on constitutive relation and processing maps focus on aluminum alloy, nickel alloy, and other alloys, with few studies focusing on 21-4N heat-resistant steel. With increasing application of 21-4N heat-resistant steel, it is necessary to carry out systematic studies on its constitutive relation and processing maps.

In this paper, based on the results of hot deformation compression test of 21-4N heat-resistant steel, the relationship between constitutive relation and hot working parameters were analyzed. Processing maps were worked out and processing stable domains and instability domains were analyzed. Finally, the optimum hot working parameter ranges were determined and verified by the material microstructure.

## 2. Materials and Experimental Procedures

The experimental material is 21-4N heat-resistant steel, and its chemical composition is shown in Table 1.

**Figure 1:**
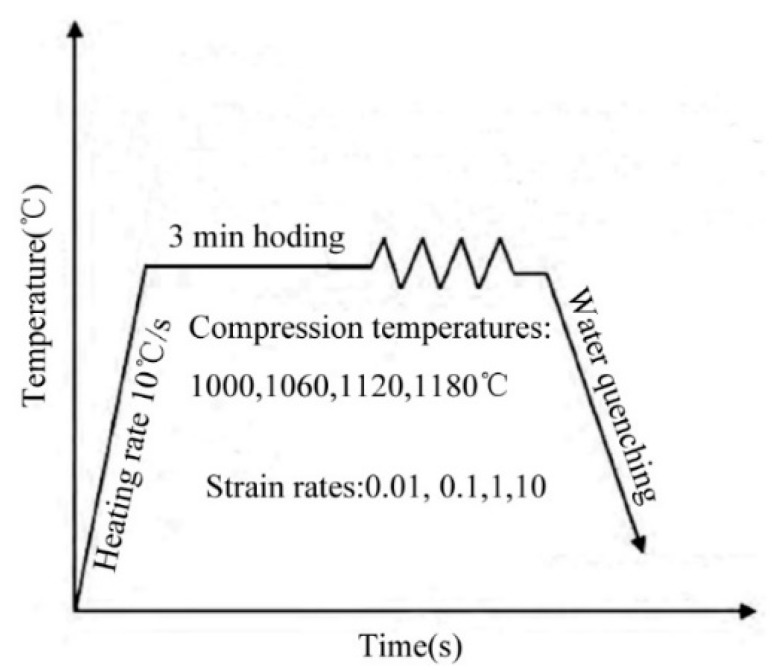
Experimental process.

The hot deformation compression test of 21-4N heat-resistant steel sample was carried out using a Gleeble-1500D thermal simulation test machine (Duffer, Durham, NC, USA). The process of unidirectional hot compression under preset hot deformation was realized by the automatic control system of the thermal simulation testing machine. The deformation temperature of the experiment was measured by thermocouple and sampled experiment under different deformation conditions by computer control system to obtain the stress and strain data. The experimental scheme of hot simulation was as Figure 1: the material was heated at rate of 10 °C/s to 1060, 1120, 1180 °C respectively, and held at that temperature for 3 min. Then, isothermal deformation compression testing was carried out under the formulate strain rates. The strain rates established in this experiment were: 0.01, 0.1, 1, 10 s^−1^, the deformation degree was 60% (the true strain was 0.916). After the hot deformation compression test, water quenching was performed immediately.

## 3. Results and Discussion

### 3.1. Flow Stress-Strain Behavior

**Figure 2:**
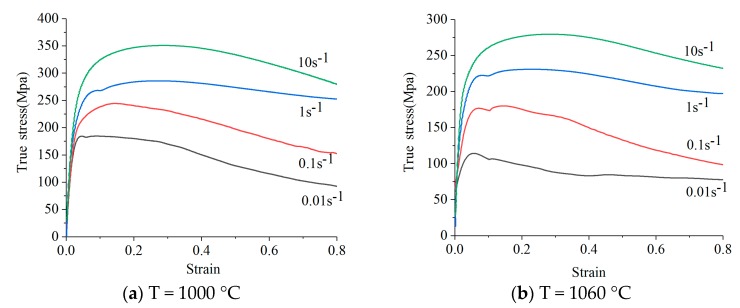

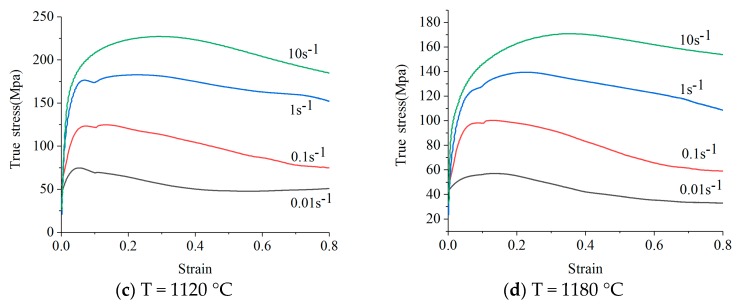
Stress-strain curves of 21-4N heat-resistant steel at different strains with temperature of (**a**) 1000 °C; (**b**) 1060 °C; (**c**) 1120 °C; (**d**) 1180 °C [33].

According to the results of the isothermal hot compression test, the real stress and strain under different strains, different deformation temperatures and different strain rates were plotted as stress-strain curves, as shown in Figure 2. Some rules are obtained concerning the rheological stress, deformation temperature, strain rates and strains based on the figures:

1. When deformation temperature is constant, the rheological stress increases with the increase in strain rate; when strain rate is constant, the rheological stress decreases with the increase in deformation temperature.
2. At the initial stage of deformation (strain at 0–0.2), the rheological stress increases rapidly and reaches the peak with the increase in strain; when strain is larger than 0.4, the rheological stress decreases with the increase in strain, and the curves present a dynamic recrystallization. Finally, it tends to be stable and enters the steady state deformation stage.
3. Stress has a significant hardening stage before reaching its peak, which is related to the increase in dislocation density and incomplete subgrain boundary.
4. Stress-strain curves are all single-peak curves; it can be concluded that the main softening mechanism of 21-4N heat-resistant steel in the high-temperature deformation process is dynamic recrystallization.

### 3.2. Constitutive Equation

The constitutive equation refers to the functional relationship between flow stress and hot working parameters (strain, deformation temperature, strain rate) during the deformation process of material, and links flow stress of material and its various hot working parameters. There exists a hot activation process during metal hot processing deformation, and the strain rate is controlled by the hot activation process. The most important data for high-temperature rheological characteristics is rheological stress, while strain rate and deformation temperature have a great influence on rheological stress. The main factors affecting rheological stress are chemical composition, technological parameters and evolution of microstructure (such as work hardening, flow softening, dynamic recrystallization, etc.) [34,35,36,37].

#### 3.2.1. Stress Parameters

The peak stress constitutive equation of 21-4N heat-resistant steel was based on the Arrhenius constitutive equation, as shown in Equation (1), which is commonly used to correlate rheological stress, strain rate and deformation temperature.

(1) $$\dot{\varepsilon }=f(\sigma )\exp(\frac{-Q}{RT})$$

In accordance with the different stress levels, the Arrhenius constitutive equation can be expressed in three forms, and the constitutive equation of 21-4N heat-resistant steel established in this paper was also based on the following three forms:

1. At a low stress level (*ασ* < 0.8), Equation (1) can be simplified into an exponential form:
  (2) $$\dot{\varepsilon }=A\sigma^{m}\exp(\frac{-Q}{RT})$$
2. At a high stress level (*ασ* > 1.2), Equation (1) can be simplified into a power exponential form:
  (3) $$\dot{\varepsilon }=B\exp(\beta \sigma )\exp(\frac{-Q}{RT})$$
3. At full stress level, Equation (1) can be simplified to hyperbolic sine form:
  (4) $$\dot{\varepsilon }=C{\left[\sinh(\alpha \sigma )\right]}^{n}\exp(\frac{-Q}{RT})$$
  where *α*, *β*, and m satisfy the relationship shown in Equation (5):
  (5) $$\alpha =\frac{\beta }{m}$$

In the equation: $\dot{\varepsilon }$—Strain rate, s^−1^; *A*, *B*, *C*—Material constants independent of temperature, s^−1^; *σ*—True stress, MPa; *R*—Gas constant, 8.3145·mol^−1^·K^−1^; *Q*—Deformation activation energy, J·mol^−1^; *T*—Temperature, °C; *m*, *n*—stress exponent; *α*, *β*—Temperature-independent stress level parameters.

Parameter *Z* (Zener-Hollomon) is used to describe the effect of strain rate and deformation temperature on rheological stress. *Z* is an exponential equation, and can be expressed as Equation (6):

(6) $$Z=\dot{\varepsilon }\cdot \exp(\frac{Q}{RT})=C{\left[\sinh(\alpha \sigma )\right]}^{n}$$

Using rheological stress data obtained by isothermal hot deformation compression test, the material constants of the constitutive equation were solved. In this paper, the peak stress for evaluating the material constant was selected, and the constitutive equations of the peak stress of 21-4N heat-resistant steel were established in different phase states. Table 2 shows the peak stress of the modified 21-4N heat-resistant steel.

*σ* was selected as peak stress, and logarithms from both sides of Equations (2) and (3) were made available:

(7) $$\ln\dot{\varepsilon }=m\ln\sigma +\ln A-\frac{Q}{RT}$$

(8) $$\ln\dot{\varepsilon }=\beta \sigma +\ln B-\frac{Q}{RT}$$

**Figure 3:**
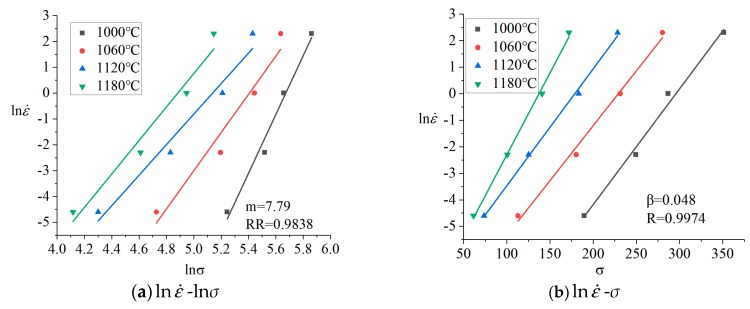
The linear relationships of (**a**) ln$\dot{\varepsilon }$ and ln*σ*; (**b**) ln$\dot{\varepsilon }$ and *σ*.

Peak stress was brought into Equations (7) and (8), then the data was subjected to linear regression fitting at different temperatures (1000, 1060, 1120, 1180 °C). Using Origin, the linear regression curves of ln$\dot{\varepsilon }$-ln*σ* and ln$\dot{\varepsilon }$-*σ* were obtained, as shown in Figure 3.

Found using Equations (7) and (8), *m* and *β* were the slopes of the ln$\dot{\varepsilon }$-ln*σ* and ln$\dot{\varepsilon }$-*σ*. Through regression processing, the following values of *m* and *β* were obtained: *m* = 7.79, *β* = 0.048.

Then, by Equation (5) *α* = *β*/*m*: *α* = 0.0062 MPa^−1^, and then the logarithm of both sides of Equation (4) was taken:

(9) $$\ln\dot{\varepsilon }=\ln C+n\ln[\sinh(\alpha \sigma )]-\frac{Q}{RT}$$

When the deformation temperature was constant:

(10) $$n=\frac{\partial \ln\dot{\varepsilon }}{\partial \ln[\sinh(\alpha \sigma )]}$$

When the strain rate was constant:

(11) $$\frac{Q}{Rn}=\frac{\partial \ln[\sinh(\alpha \sigma )]}{\partial (1/T)}$$

**Figure 4:**
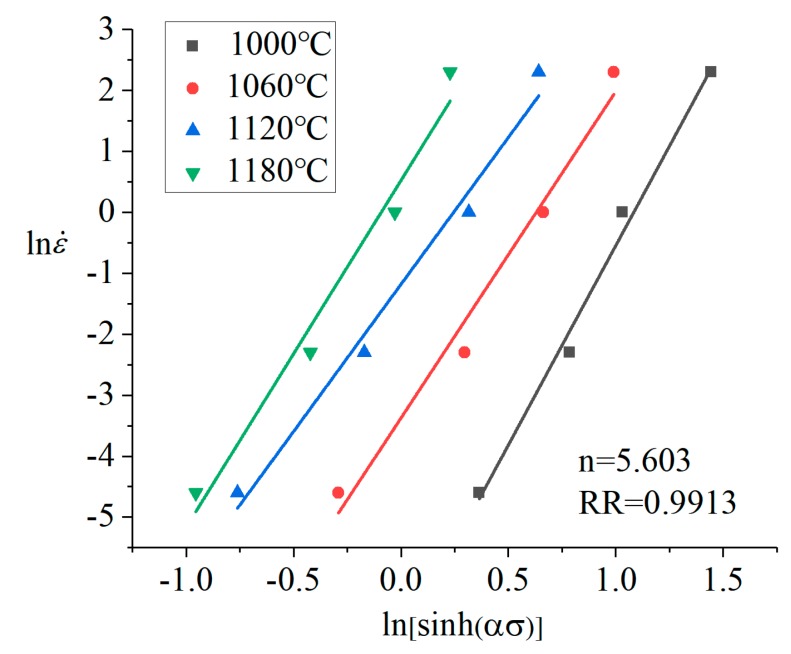
The linear relationships of ln$\dot{\varepsilon }$ and ln[sinh(*ασ*)].

*α* was brought into Equation (10), then the data was subjected to linear regression fitting at different temperatures (1000, 1060, 1120, 1180 °C) by Origin. The linear regression curve of ln$\dot{\varepsilon }$-ln[sinh(*ασ*)] was obtained, as shown in Figure 4. The stress index can be obtained by using Equation (10): *n* = 5.603.

**Figure 5:**
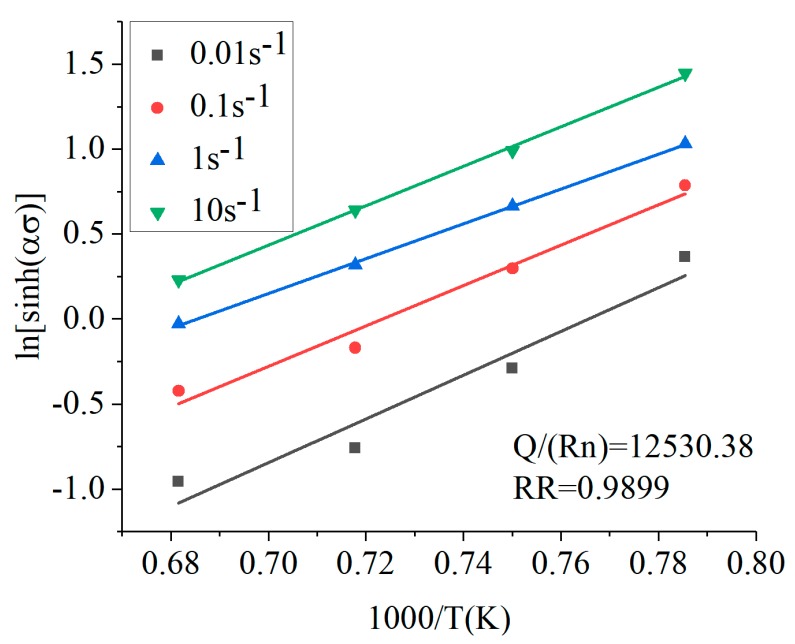
The linear relationships of 1000/T and ln[sinh(*ασ*)].

α was brought into Equation (11), then the data was subjected to linear regression fitting at different temperatures (1000, 1060, 1120, 1180 °C). The linear regression curve of ln[sinh(*ασ*)]-1000/T was obtained, as shown in Figure 5. Combining Equations (9) and (11), it can be concluded that the slope of the curve was *Q*/*Rn*.

Assuming *R* = 8.3145·mol^−1^·K^−1^ and the average of the curve slope as calculated by *n*, the deformation activation energy was obtained: *Q* = 583.742 KJ·mol^−1^.

In this paper, hyperbolic sinusoidal function was used to describe the peak stress constitutive equation of 21-4N heat-resistant steel. After calculation, the Zener-Hollomon parameter expression of 21-4N heat-resistant steel was obtained as follows:

(12) $$Z=\dot{\varepsilon }\cdot \exp(\frac{Q}{RT})=\dot{\varepsilon }\cdot \exp\frac{583742}{8.3145T}$$

The value of *Z* at different deformation temperatures and strain rates can be obtained from Equation (12). Assuming the logarithm of both sides of Equation (6):

(13) lnZ=lnC+nln[sinh(ασ)]

**Figure 6:**
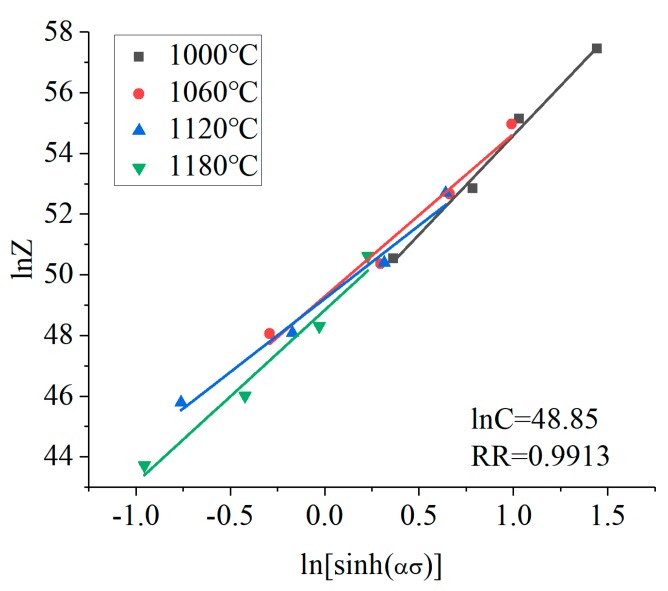
The linear relationships of ln[sinh(*ασ*)] and ln*Z*.

From Equation (13), ln[sinh(*ασ*)] and ln*Z* were linear, and the data was subjected to linear regression fitting at different temperatures (1000, 1060, 1120, 1180 °C). Then the linear regression curve of ln*Z*-ln[sinh(*ασ*)] was obtained, as shown in Figure 6. Combined with Equation (13), it can be concluded that ln*C* was the intercept of the curve. The average intercept was obtained by fitted the curve: ln*C* = 48.85, *C* = 1.645 × 10^21^.

According to the properties of the hyperbolic sine function, Equation (13) can be rewritten as:

(14) $${\left(\frac{Z}{C}\right)}^{\frac{1}{n}}=\sinh(\alpha \sigma )=(e^{\alpha \sigma }-e^{-\alpha \sigma })/2$$

Then:

(15) $$\sigma =\frac{1}{\alpha }\ln({\left(\frac{Z}{C}\right)}^{\frac{1}{n}}+{\left({\left(\frac{Z}{C}\right)}^{\frac{2}{n}}+1\right)}^{\frac{1}{2}})$$

Taking *C*, *α*, *n*, *Q*, into Equation (15) and combining with Equation (12), the peak stress constitutive equation of 21-4N can be obtained:

(16) $$\dot{\varepsilon }=1.64\times 10^{21}{\left[\sinh(0.0062\sigma )\right]}^{5.603}\exp(\frac{-583742}{8.3145T})$$

In the equation: $\dot{\varepsilon }$—Strain rate, s^−1^; *σ*—True flow stress, MPa; *T*—Temperature, °C.

#### 3.2.2. Accuracy Verification of Constitutive Equation

To evaluate the accuracy of the peak stress constitutive equation, the average relative error *AARE* and the correlation coefficient *RR* were introduced; the expressions were as shown in Equations (17) and (18). The smaller relative error *AARE* and the closer correlation coefficient *RR* were equal to 1, establishing the higher accuracy of constitutive equation as the more accurate prediction.

(17) $$AARE(\%)=\frac{1}{N}\sum_{i=1}^{N}\left|\frac{E_{i}-P_{i}}{E_{i}}\right|\times 100\%$$

(18) $$RR=\frac{\sum_{i=1}^{n}\left(E_{i}-\bar{E})(P_{i}-\bar{P}\right)}{\sqrt{{\sum_{i=1}^{n}\left(E_{i}-\bar{E}\right)}^{2}{\left(P_{i}-\bar{P}\right)}^{2}}}$$

In the equation: *E*—Peak stress experimental value; *P*—Peak stress prediction value (calculated value); $\bar{E},\bar{P}$—Average value of peak stress experimental value and peak stress prediction value.

Equation (12) was used to calculate *Z* at different temperatures and strain rates. Then, the results were substituted into peak stress constitutive Equation (16), and the corresponding rheological stress was calculated, as shown in Table 3.

The calculated rheological stress was compared with corrected rheological stress (Table 2) to find relative error, as shown in Table 4.

**Figure 7:**
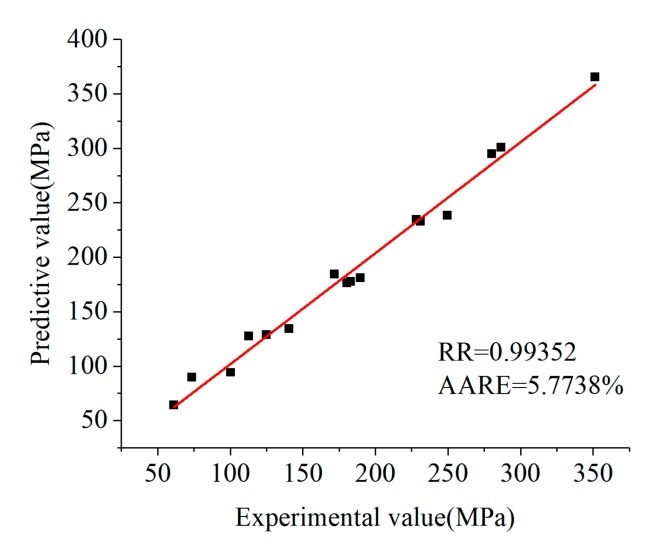
The correlation between experimental stress and predicted stress of the peak stress of 21-4N heat-resistant steel.

The correlation between experimental stress and predicted stress (calculated stress) of peak stress is shown in Figure 7. The data in Table 4 were calculated by using Equation (18), and then the average relative error and the correlation coefficient were obtained: *AARE* = 5.9463%; *RR* = 0.99343. This indicates that the peak stress constitutive equation established in this paper can well describe the flow stress relationship of 21-4N heat-resistant steel at different temperatures and strain rates.

### 3.3. Processing Map

Processing maps can reflect the influence of hot working parameters on the microstructure of materials, can simulate intrinsic deformation behavior of materials, and can reveal stable processing domains with good structures and non-stable processing domains with tissue defects. It is a superposition of the power dissipation map and the destabilization map [38,39,40].

At present, the most widely used hot deformation model for metal materials is the Dynamic Material Model (DMM) [41,42,43,44]. The basic principle of it is: assume that the equipment, mold and sample are a thermodynamically closed system; the hot deformation process is related to energy dissipation. The total energy input (*P*) of the sample is consumed in two ways: one is power dissipation caused by the plastic deformation of the sample, which is called dissipation (*G*); the other is power dissipation caused by the structural changes taking place during the plastic deformation process of the sample (such as dynamic recovery, dynamic recrystallization, hole formation, wedge crack formation, dissolution and growth of particles or phases under dynamic conditions and phase change or precipitation induced by deformation, and so on), which is called dissipation of quantity (*J*). As shown in Equation (19):

(19) $$P=\dot{\varepsilon }\cdot \sigma =G+J=\int_{0}^{\dot{\varepsilon }}\sigma d\dot{\varepsilon }+\int_{0}^{\sigma }\dot{\varepsilon }d\sigma$$

In the equation: Dissipation $G=\int_{0}^{\dot{\varepsilon }}\sigma d\dot{\varepsilon }$; Dissipation of quantity $J=\int_{0}^{\sigma }\dot{\varepsilon }d\sigma$. The proportions of *G* and *J* are determined by strain rate sensitivity (*m*), the expression of which is shown as Equation (20):

(20) $$m={\frac{\partial (\mathrm{lg}\sigma )}{\partial (\mathrm{lg}\dot{\varepsilon })}|}_{\varepsilon ,T}$$

The efficiency of power dissipation (*η*) is a function of m, the relationship is shown as Equation (21):

(21) $$\eta =\frac{2m}{m+1}$$

In this paper, the Prasad criterion instability criterion is adopted, and its expression is shown as Equation (22):

(22) $$\xi (\dot{\varepsilon })=\frac{\partial [\ln(\frac{m}{m+1})]}{\partial (\ln\dot{\varepsilon })}+m<0$$

#### 3.3.1. Processing Map of 21-4N Heat-Resistant Steel

Drawing process of processing maps of 21-4N heat-resistant steel was as follows:

1. The stress under the conditions of strains of 0.2, 0.4 and 0.6 were respectively obtained from the stress-strain curves, as shown in Table 5.
2. The cubic spline interpolation function was used to interpolate ln*σ*-ln$\dot{\varepsilon }$ at different deformation temperatures, and the strain rate sensitivity (*m*) of each node at different deformation temperatures was obtained. The results are shown in Table 6.
3. Equations (3) and (4) were used to calculate the efficiency of the power dissipation (*η*) of each node at different deformation temperatures.
4. The cubic spline interpolation function was used to interpolate ln(m/(m + 1)) − ln$\dot{\varepsilon }$ at different deformation temperatures, and the criteria of each node were obtained.
5. Energy dissipation maps and destabilization maps were drawn, and they were overlaid to obtain the processing maps of 21-4N heat-resistant steel.

#### 3.3.2. Analysis of Processing Map of 21-4N Heat-Resistant Steel

**Figure 8:**
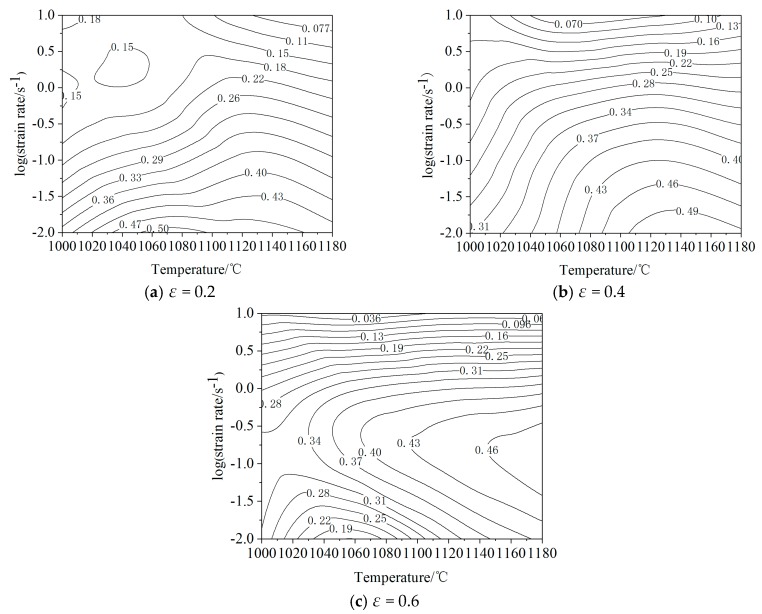
Energy dissipation map of 21-4N heat-resistant steel with strain of (**a**) 0.2; (**b**) 0.4; (**c**) 0.6.

Figure 8 shows the energy dissipation maps of 21-4N heat-resistant steel at strains of 0.2, 0.4, and 0.6. It can be seen from the figure that: the contour line represents the efficiency of power dissipation (*η*); the power dissipation increases gradually from the upper-left corner to the bottom right; the power dissipation increases with the increase in deformation temperature and the decrease in strain rate; the peak value of power dissipation shows little change with the increase in strain, indicating that the hot deformation process of 21-4N heat-resistant steel basically enters a steady-state deformation stage after the strain variable reaches 0.4, and rheological stress is relatively stable. When the strain is 0.2 (Figure 8a), *η* reaches its peak at a deformation temperature of 1060–1120 °C and a strain rate of 0.01–0.1s^−1^, which is about 0.50. When the strain is 0.4 (Figure 8b), *η* reaches its peak at a deformation temperature of 1120 –1180 °C and a strain rate of 0.01–0.1 s^−1^, which is about 0.49. When the strain is 0.6 (Figure 8c), *η* reaches its peak at a deformation temperature of 1120–1180 °C and a strain rate of 0.01–1 s^−1^, which is about 0.46. Studies have shown that the larger the value of *η*, the better the machinability of the material, and the deformation domain may be in the best condition for the hot deformation process [45,46,47].

**Figure 9:**
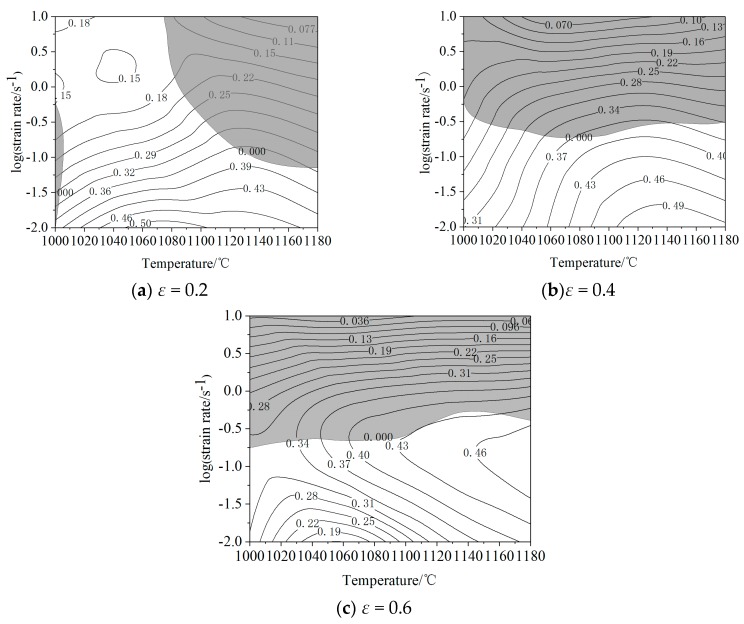
Processing map of 21-4N heat-resistant steel with strains of (**a**) 0.2; (**b**) 0.4; (**c**) 0.6.

Figure 9 shows the processing maps of 21-4N heat-resistant steel under strains of 0.2, 0.4 and 0.6. Figures are clearly divided into two parts: the shadow domain is the processing instability domain, and the non-shadow domain is the stable processing domain. The instability domain is mainly located at high strain rate. In processing maps, domains with η values greater than 0.3 are usually taken to be high-power dissipation domains; this domain is regarded as resulting in the best hot deformation properties of metal materials [48]. The value of η in the stable domain is basically bigger than 0.3, which indicates that the energy dissipation during the deformation process is lower, which is beneficial to hot deformation. The maximum values of *η* appear below the right of the safe domain, in which the deformation is 1060–1180 °C, and the strain rate is 0.01–0.1 s^−1^. According to stress-strain curves, dynamic recovery and dynamic recrystallization occur frequently in this domain, indicating that the materials in this domain have good hot processing performance. The instability domain is concentrated in the upper part, in which the strain rate is 1–10 s^−1^ and *η* is at its minimum, and is not suitable for hot processing.

Based on the processing maps, the hot working process of 21-4N heat-resistant steel can be obtained: the domain suitable for deformation has a temperature of 1000–1180 °C and a strain rate of 0.01–0.1 s^−1^. Optimum hot working parameters: deformation temperature of 1120–1180 °C, strain rate of 0.01–0.1 s^−1^. In this domain, η can reach more than 40% and 21-4N has good processing performance. When the deformation temperature is 1000–1120 °C and strain rate is 1–10 s^−1^, *η* is relatively small, and the 21-4N has poor machining performance. Therefore, it is necessary to keep away from this domain and the corresponding processing parameters during practical application.

### 3.4. Microstructural Observation

**Figure 10:**
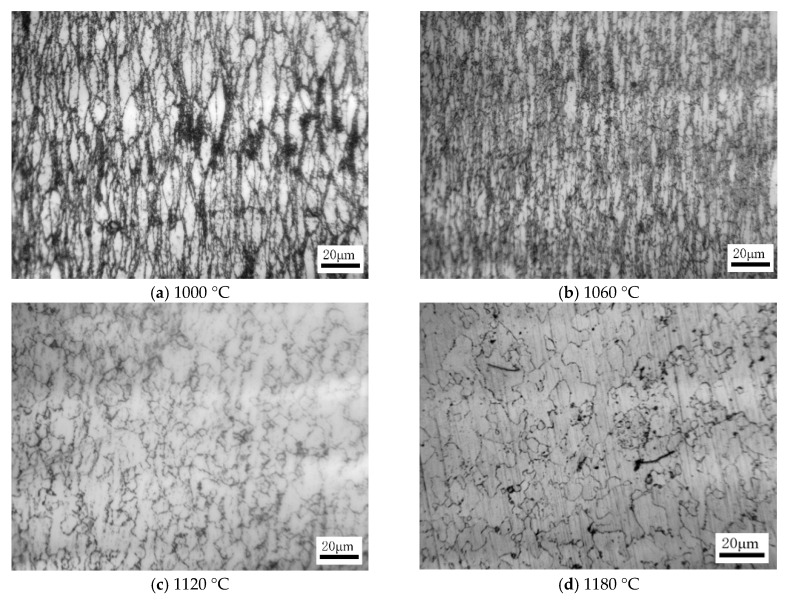
The microstructures of the specimens deformed at: (**a**) 1000 °C, *ε* = 0.6, $\dot{\varepsilon }$ = 0.1 s^−1^; (**b**) 1060 °C, *ε* = 0.6, = 0.1 s^−1^; (**c**) 1120 °C, *ε* = 0.6, $\dot{\varepsilon }$ = 0.1 s^−1^; (**d**) 1180 °C, *ε* = 0.6, $\dot{\varepsilon }$ = 0.1 s^−1^.

In the processing maps of 21-4N heat-resistant steel, the microstructure under different deformation conditions is different. To fully understand the deformation process of 21-4N heat-resistant steel, it is necessary to study the microstructure of different deformation regions. Take the processing map with a strain of 0.6 as an example to analyze the microstructure evolution law of the sample. When *ε* = 0.6, $\dot{\varepsilon }$ = 0.1 s^−1^, the microstructure of 21-4N after deformation at the indicated temperatures is shown in Figure 10. When the deformation temperature is 1000 °C, its power dissipation coefficient is 0.19–0.31. Some grains reach the dynamic recrystallization nucleation energy and begin to nucleate and grow around the original grain boundary, and dynamic recrystallization occurs (Figure 10a). When the temperature rises to 1060 °C, the number of dynamically recrystallized grains gradually increases, and the original grains are severely elongated (Figure 10b). When the temperature rises to 1120 °C, the power dissipation coefficient reaches 0.40–0.43. Most grains have completed dynamic recrystallization, only a small number of deformed original grains exist (Figure 10c). When the temperature rises to 1180 °C, the power dissipation coefficient reaches 0.43–0.46; the dynamic recrystallization is basically completed, and the grains have been roughened (Figure 10d). According to the above analysis, the processing maps of 21-4N heat-resistant steel play an important guiding role in predicting the microstructure of hot deformation.

## 4. Conclusions

The hot deformation characteristics of 21-4N were evaluated by high-temperature compression test in this study. The following conclusions were drawn from the main results:

1. During hot deformation of 21-4N heat-resistant steel, rheological stresses decrease with increasing temperature and decreasing strain rate; rheological stress curves show obvious dynamic recrystallization at high temperature and low strain rate; with decreasing temperature and strain rate, rheological stress curves present obvious dynamic recovery; due to work hardening, stress quickly reaches peak, and then gradually drops to a steady rheological stress under the action of dynamic recrystallization, and enters the stable deformation stage.
2. The hot deformation activation energy of 21-4N heat-resistant steel is calculated to be 583.742 KJ·mol^−1^. Based on the hyperbolic sine equation, the relationship among the peak stress, deformation temperature and strain rate can be described by the constitutive equation as follows:
  (23) $$\dot{\varepsilon }=1.64\times 10^{21}{\left[\sinh(0.0062\sigma )\right]}^{5.603}\exp(\frac{-583742}{8.3145T})$$
3. The favorable hot deformation conditions are located in one domain of the processing maps, with a peak efficiency of more than 0.4 occurring at a temperature of about 1120–1180 °C and a strain rate of 0.01–0.1 s^−1^.

## Figures and Tables

**Table 1:** Chemical composition of the 21-4N sheet (wt.%).

C	Si	Mn	S	P	Cr	Ni	N	Fe
0.48–0.58	≤0.35	8–10	≤0.03	≤0.04	20–22	3.25–4.5	0.35–0.5	Bal.

**Table 2:** Peak stress of the modified 21-4N heat-resistant steel.

Deformation Degree (%)	T/°C	Strain Rate/s^−1^	Peak Stress/MPa
60	1000	0.01	189.6
		0.1	249.6
		1	286.8
		10	351.5
	1060	0.01	112.9
		0.1	180.5
		1	231.4
		10	280.3
	1120	0.01	73.7
		0.1	125.1
		1	183.1
		10	228.3
	1180	0.01	61.3
		0.1	100.5
		1	140.7
		10	171.8

**Table 3:** The calculated rheological stress (*σ*) of 21-4N steel under different hot deformation conditions (MPa).

$\dot{\varepsilon }$/s^−1^	*T*/°C			
	1000	1060	1120	1180
0.01	181.2	128.2	90.0	63.6
0.1	238.6	177.0	128.9	93.2
1	300.8	233.9	177.9	133.2
10	365.7	295.9	234.8	183.0

**Table 4:** The relative error (*ARRE*) between theoretical and corrected experimental.

$\dot{\varepsilon }$/s^−1^	*T*/°C			
	1000	1060	1120	1180
0.01	0.0444	0.1353	0.2203	0.0376
0.1	0.0442	0.0192	0.0302	0.0723
1	0.0488	0.0110	0.0288	0.0535
10	0.0403	0.0554	0.0288	0.0652

**Table 5:** Stress (*σ*/MPa) under different deformation conditions.

Strain	*T*/°C	$\dot{\varepsilon }$/s^−1^			
		0.01	0.1	1	10
0.2	1000	155.4	229.1	282.5	346.0
	1060	98.1	174.5	230.5	278.0
	1120	63.9	119.9	182.3	224.1
	1180	55.3	97.1	138.4	161.0
0.4	1000	150.4	215.8	281.8	346.2
	1060	82.7	145.0	225.2	276.4
	1120	51.2	104.4	175.5	223.7
	1180	42.7	83.1	132.6	170.4
0.6	1000	115.7	179.4	265.9	321.0
	1060	81.4	120.1	201.8	262.8
	1120	48.8	86.1	157.6	214.3
	1180	34.1	67.1	129.5	178.0

**Table 6:** The value of *m* of each computing node at different deformation temperatures.

**Strain**	*T*/°C	$\dot{\varepsilon }$/s^−1^			
		0.01	0.1	1	10
0.2	1000	0.232	0.117	0.077	0.111
	1060	0.345	0.171	0.086	0.091
	1120	0.323	0.229	0.136	0.044
	1180	0.290	0.199	0.110	0.022
0.4	1000	0.182	0.134	0.100	0.081
	1060	0.254	0.226	0.148	0.022
	1120	0.340	0.274	0.171	0.033
	1180	0.330	0.247	0.157	0.060
0.6	1000	0.177	0.192	0.138	0.014
	1060	0.085	0.225	0.198	0.003
	1120	0.190	0.279	0.222	0.021
	1180	0.252	0.313	0.235	0.019
